# Constructing *Z*‑Scheme Ni-MOF-74/CoAl-Layered Double Hydroxide Heterojunctions for Enhanced Photocatalytic CO_2_ Reduction

**DOI:** 10.1021/acsami.5c23229

**Published:** 2026-02-17

**Authors:** Can Wang, Zhiyao Wu, Mengwei Chen, Yuxiang Deng, Guilin He, Xinpeng Wang, Yanqiu Zhu, Nannan Wang

**Affiliations:** † State Key Laboratory of Featured Metal Materials and Life-cycle Safety for Composite Structures, MOE Key Laboratory of New Processing Technology for Nonferrous Metals and Materials, and School of Resources, Environment and Materials, 12664 Guangxi University, Nanning 530004 China; ‡ State Key Laboratory of Chemistry for NBC Hazards Protection, Frontiers Science Center for Rare Isotopes, School of Nuclear Science and Technology, 12426 Lanzhou University, Lanzhou 730000 China; § Faculty of Environment, Science and Economy, 3286 University of Exeter, Exeter EX4 4QF U.K.

**Keywords:** photocatalytic CO_2_ reduction, *Z*-scheme heterojunction, band structure

## Abstract

Constructing *Z*-scheme heterojunctions is crucial for improving the charge localization on the surface of photocatalysts and enhancing photocatalytic reduction performance. Herein, this research proposes a heterostructure construction strategy that utilizes a Nickel-based metal organic framework with MOF-74 topology (Ni-MOF-74) as a structural template for deriving ultrathin CoAl-LDH nanosheets (denoted as 20-NiL). This approach enables precise control over the two-dimensional lamellar morphology and interfacial electronic structure, facilitating electron–hole pair separation and mitigating CoAl-LDH nanosheet aggregation. Under simulated solar irradiation, 20-NiL exhibits a CO production rate of 79.86 μmol·g^–1^·h^–1^, representing a 70% enhancement over the pristine components. By comparing the XPS spectra before and after the photocatalytic reaction, we confirm the charge transfer mechanism of the *Z*-scheme heterojunction: the binding energies of Co and Al increase, while that of Ni decreases, indicating the transfer of electrons (e^–^) from CoAl-LDH to Ni-MOF-74 upon light irradiation. In situ Fourier transform infrared spectroscopy combined with Soft X-ray absorption spectroscopy elucidates the 2e^–^ pathway for CO_2_ conversion to CO through the dominant intermediates COOH* and CO*. This work is expected to provide helpful reference for the development of *Z*-scheme heterojunction photocatalysts and the investigation of their charge transfer kinetics.

## Graphical abstract

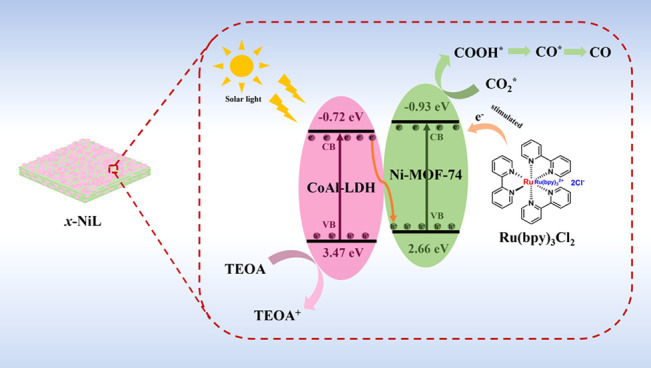

## 1 Introduction

With the acceleration of industrialization and the excessive consumption of fossil fuels, the concentration of CO_2_ in the atmosphere continues to rise, triggering severe environmental issues such as global warming and ocean acidification. To achieve the “carbon neutrality” goal, photocatalytic technology that utilizes solar energy to convert CO_2_ into carbon-based fuels (e.g., CO, CH_4_) has emerged as a research hotspot, owing to its dual advantages of environmental remediation and energy regeneration. However, the inherent chemical inertness of the CO_2_ molecule (with a C=O bond dissociation energy as high as 750 kJ/mol), coupled with the kinetic barriers of the multielectron reduction process, severely restricts its conversion efficiency.^1^ Particularly, the CO generation pathway requires the transfer of 2 electrons and 2 protons, while the CH_4_ generation pathway involves an 8-electron and 8-proton transfer process, placing extremely high demands on the charge separation and transport capabilities of the photocatalytic system.^2^

Layered double hydroxides (LDHs), a class of two-dimensional layered composite metal hydroxides consisting of divalent and trivalent metal cations, have garnered significant attention in the field of photocatalysis, particularly for photocatalytic CO_2_ reduction, due to their tunable chemical composition and structural features. The core advantages of LDHs include the highly uniform distribution of metal cations, exchangeable interlayer anions, good visible-light response, strongly basic surface properties, and excellent CO_2_ adsorption capacity.^3^ However, despite their considerable potential for photocatalytic CO_2_ reduction, LDHs synthesized by conventional methods often suffer from particle aggregation leading to reduced specific surface area, limited light absorption capability, and universally face the critical bottleneck of rapid recombination of photogenerated charge carriers, which constraints their photocatalytic activity. In order to overcome these limitations, constructing heterojunctions between LDHs and suitable semiconductors has emerged as an effective strategy for performance enhancement. Among various LDHs, CoAl-LDH is regarded as an excellent candidate for photocatalytic CO_2_ reduction reaction (PCO_2_RR) due to its unique layered structure, broad-spectrum solar light response, flower-like hierarchical morphology (providing high specific surface area and abundantly exposed active sites), inherent basic surface (enhancing CO_2_ adsorption), and uniformly distributed reductive Co^2+^ active sites on the layers.^4^ Recent research has focused on developing heterojunction photocatalysts based on CoAl-LDH, such as the *Z*-scheme CoAl-LDH/InVO_4_-30 exhibited a CO production rate 2.46 times higher than that of pure CoAl-LDH.^5^ A ternary *Z*-scheme CoAl-LDH/CeO_2_/RGO system achieved a CO generation rate of 5.5 μmol·g^–1^·h^–1^ by synergistically accelerating charge transfer, enhancing light harvesting, and improving photon utilization efficiency.^6^ The type-II CoAl-LDH@Cu_2_O system constructed a p–n heterojunction with a strong built-in electric field, significantly promoting electron transfer and separation capabilities.^1^ Other systems, such as ZnAl-LDH/ZIF-8 and TiMgAl-LDH/GO, also demonstrated promising performance owing to enhanced charge carrier separation efficiency.^7,8^ Given the unique advantages of CoAl-LDH and the notable achievements with various heterojunctions, this study focuses on designing novel and efficient heterojunction systems based on CoAl-LDH. It aims to optimize synthesis strategies to control morphology, suppress aggregation, and further enhance the performance of light-driven CO_2_ conversion.

Metal organic frameworks (MOFs), a class of highly porous crystalline materials formed by the self-assembly of organic ligands and metal ions,^9^ are theoretically suitable for photocatalytic CO_2_ reduction due to their unique electronic structures and diverse morphologies (e.g., rhombic polyhedral structures), having also demonstrated significant potential particularly in photocatalytic water splitting for hydrogen production. However, the practical application of MOFs faces notable challenges: their inherent wide bandgap restricts efficient absorption of the solar spectrum, especially visible light; concurrently, low electron transfer rates and quantum efficiency severely limit their photocatalytic activity. Furthermore, at a CO_2_ concentration of 0.1%, the CO generation rate of the Ni-MOF monolayer is only 3–58 μmol·h^–1^·g^–1^.^10^ To overcome these limitations, developing heterojunction materials based on MOF precursors has emerged as a reliable strategy for enhancing photocatalytic performance. For instance, Li et al.^11^ grew nanoparticle Ni_2_P and peanut-like BiVO_4_ on a rhombic-structured Ni-MOF-74 substrate, which not only increased active sites but also captured more protons for hydrogen evolution. Yao et al.^12^ fabricated a ZnIn_2_S_4_/P–Ni-MOF-74 heterojunction via in situ phosphidation; its heterogeneous interface and the unique morphology derived from the MOF provided abundant active sites, significantly accelerating electron transfer. Dong et al.^13^ successfully modified Ni-MOF-74 material through temperature and solvent modulation, achieving a highly porous structure (NI-74-AM) that enhanced performance for photocatalytic CO_2_ conversion. These approaches not only mitigate the intrinsic limitations of MOFs but also create new paradigms for designing highly efficient and stable photocatalysts.

It is noteworthy that, inspired by Feng et al.^14^ and Zhao et al.,^15^ MOFs can serve as functional templates or substrates for the controllable synthesis of layered double hydroxides (LDHs). This strategy can effectively circumvent the issue of particle aggregation commonly encountered in conventional LDH synthesis, promoting the formation of LDHs with ultrathin two-dimensional nanosheet structures and abundant active sites. These structural characteristics are conducive to modulating the band structure and electronic properties of the material, thereby enhancing the separation efficiency of photogenerated electron–hole pairs. Precise control over morphology and structure has been widely demonstrated to be a critical factor in improving the performance of photocatalysts, including the efficiency of light energy capture and utilization.^16^

Based on the aforementioned research, this paper proposes a derivatization strategy employing a hydrothermal synthesis method to utilize Ni-MOF as a precursor for preparing a photocatalyst. The strategy involves the templated growth of ultrathin two-dimensional CoAl-LDH nanosheets, which avoids the aggregation issue common in traditional LDH synthesis, resulting in a 63% increase in specific surface area and overcoming the limitations of single-component MOF materials. Furthermore, the incorporation of vacancy structures serves to modulate the band gap and promote charge carrier separation. Concurrently, a multiscale characterization approach combining in situ FTIR, synchrotron-based soft X-ray absorption spectroscopy (sXAS), and photoelectrochemical measurements was employed to elucidate the role of interfacial chemical bonds in facilitating charge separation within the *Z*-scheme *x*-NiL catalyst. The constructed composite system provides an effective pathway for charge transfer, thereby significantly reducing the charge transfer energy barrier. This leads to a shortened fluorescence lifetime of 0.649 ns and synergistically optimizes CO_2_ adsorption and activation capabilities, ultimately achieving a CO production rate nearly 70% higher than that of the single-component material.

## 2 Experimental Section

### 2.1 Synthesis of the Ni-MOF-74 Catalyst

First, 20 mL of DMF, deionized water, and ethanol were mixed and stirred for 15 min. Subsequently, 0.88 g of nickel nitrate hexahydrate, 0.24 g of terephthalic acid (H_2_BDC), and 1.20 g of polyvinylpyrrolidone K30 (PVP-K30) were added to the aforementioned mixed solution, followed by stirring uniformly at 500 rpm for 1 h. The resulting solution was then transferred into a 100 mL Teflon-lined stainless-steel autoclave, which was maintained at 150 °C for 10 h. After the hydrothermal reaction, the obtained green precipitate was washed via suction filtration using anhydrous ethanol and deionized water. Finally, the precipitate was dried in a vacuum oven at 60 °C for 12 h to obtain the Ni-MOF-74 green powder.

### 2.2 Synthesis of the *x*-NiL Catalyst

First, 1.5 mmol of cobalt nitrate hexahydrate, 0.5 mmol of aluminum nitrate nonahydrate, 5 mmol of urea, 2 mmol of ammonium fluoride, and 60 mL of deionized water were mixed and stirred for 20 min. Subsequently, 0.0138, 0.0311, and 0.0532 g of the as-prepared Ni-MOF-74 green powder were dispersed into the above mixture, respectively, followed by stirring uniformly at 500 rpm for 1 h. The resulting solution was then transferred into a 100 mL Teflon-lined stainless-steel autoclave, which was maintained at 100 °C for 24 h. After the hydrothermal reaction, the obtained green precipitate with a pinkish tint was washed via suction filtration using anhydrous ethanol and deionized water. Finally, the precipitate was dried in a vacuum oven at 60 °C for 12 h to obtain the final samples, designated as *x*-NiL (*x*-Ni-MOF-74/CoAl-LDH), where *x* represents the mass percentage of the added Ni-MOF-74 relative to the total mass of the final sample. Accordingly, the resulting final samples were labeled as 10-NiL, 20-NiL, and 30-NiL, respectively. The synthesis process of the *x*-NiL catalyst described in this work is illustrated in Figure 1a.

**Figure 1:**
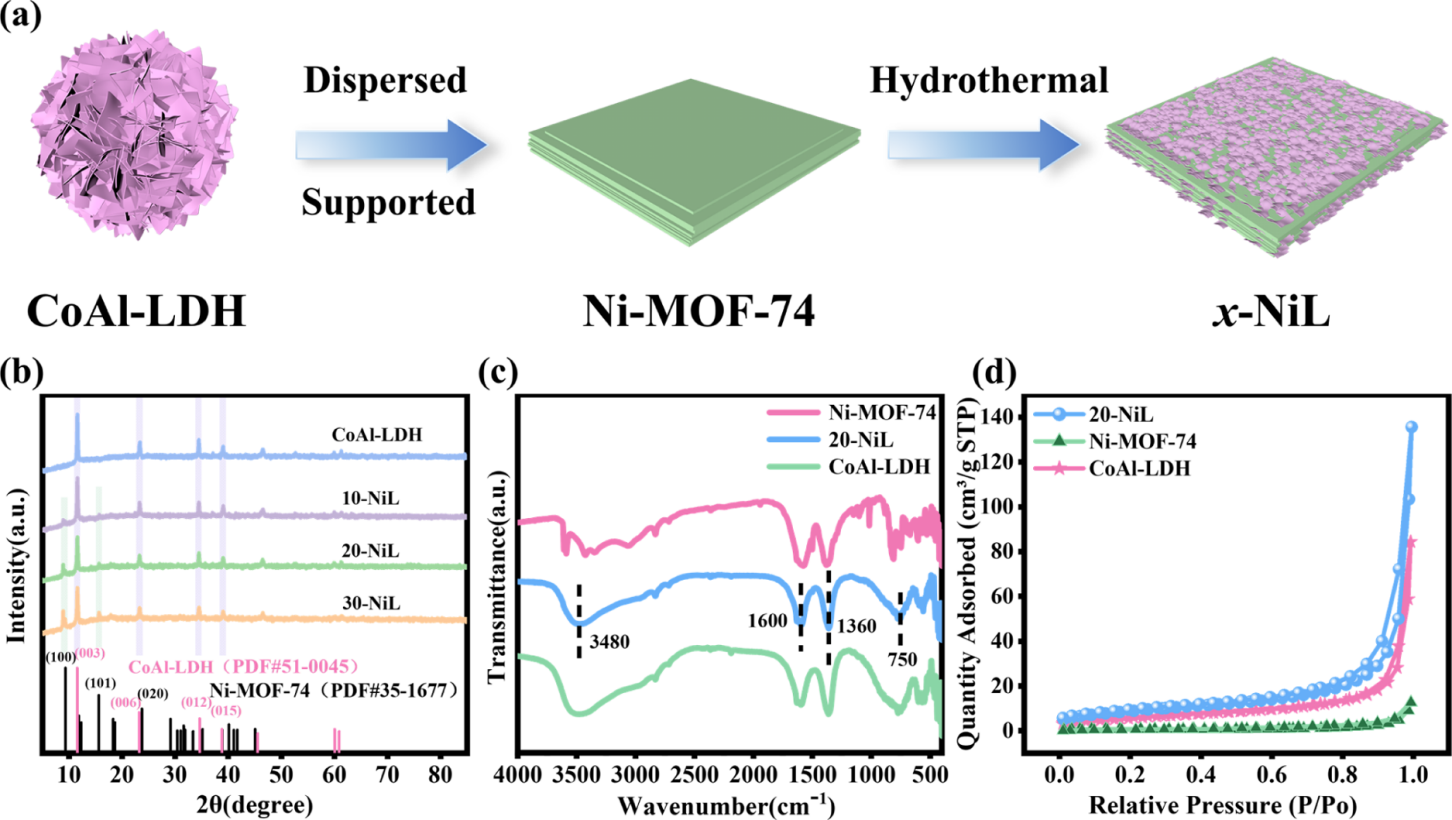
(a) Schematic illustration of the synthesis process for the composite catalyst *x*-NiL. (b) XRD patterns. (c) FT–IR spectra. (d) N_2_ adsorption–desorption isotherms of Ni-MOF-74, 20-NiL, and CoAl-LDH.

### 2.3 Catalyst Characterization and Performance Measurements

The detailed experimental methods for materials characterization, photoelectrochemical measurements, and photocatalytic CO_2_ reduction tests are provided in Text. S1 to Text. S3 (Supporting Information).

## 3 Results and Discussion

### 3.1 Morphology and Surface Chemical State

The crystal structures of the photocatalysts were investigated using X-ray diffraction (XRD), as shown in Figure 1b. According to the reference pattern for Ni-MOF-74 (PDF#35-1677), distinct diffraction peaks were observed at 2θ = 9.3°, 15.8°, and 23.8°, corresponding to the (100), (101), (020) crystal planes of Ni-MOF-74, respectively.^17^ For CoAl-LDH (PDF#51-0045), characteristic diffraction peaks were identified at 2θ = 11.5°, 23.2°, 34.6°, and 38.7°, which can be indexed to the (003), (006), (012), and (015) planes of CoAl-LDH, respectively.^18^ Furthermore, the XRD patterns of the composite *x*-NiL catalysts clearly exhibit characteristic peaks attributable to both Ni-MOF-74 and CoAl-LDH, confirming the successful synthesis of Ni-MOF-74, CoAl-LDH, and the *x*-NiL composites. Fourier transform infrared (FT–IR) spectroscopy was employed to analyze the chemical bonds and surface functional groups of the catalysts. As presented in Figure 1c, the absorption band observed around 3480 cm^–1^ is assigned to the O–H stretching vibration of the CoAl-LDH nanosheets and interlayer water molecules.^19^ The band near 1600 cm^–1^ corresponds to the bending vibration of interlayer water molecules. The peak around 1360 cm^–1^ is attributed to the ν_3_ asymmetric stretching vibration of carbonate ions (CO_3_^2–^). The band located near 750 cm^–1^ is associated with the ν_2_ planar bending vibration of M–OH and M–O bonds (where M represents metal ions).^20^ The Brunauer–Emmett–Teller (BET) specific surface areas were determined from N_2_ adsorption–desorption isotherms (Figure 1d). The 20-NiL sample exhibits a larger BET surface area (*S*_BET_ = 35.5 m^2^·g^–1^) compared to that of CoAl-LDH (*S*_BET_ = 21.82 m^2^·g^–1^) and Ni-MOF-74 (*S*_BET_ = 2.95 m^2^·g^–1^). Combined with the data on specific surface area, pore volume, and pore size of the synthesized samples (Table S1, Supporting Information), the results indicate that 20-NiL possesses a greater number of active sites, which can adsorb more reactant molecules, enhance CO_2_ mass transfer efficiency, and improve surface reaction rates. These properties are conducive to promoting the effective progression of the photocatalytic CO_2_ reduction reaction in the liquid–solid system.

The surface morphology and microstructure of the as-prepared Ni-MOF-74, CoAl-LDH, and 20-NiL samples were characterized using scanning electron microscopy (SEM) and transmission electron microscopy (TEM). As shown in Figure 2a,d, the pristine Ni-MOF-74 exhibits a polyhedral morphology composed of stacked rhombic sheets, with a length and width of approximately 12 μm.^21^ This three-dimensional structure provides ample space for supporting other materials. As depicted in Figure 2b,e, the pristine CoAl-LDH displays a three-dimensional flower-like spherical architecture assembled from numerous two-dimensional nanosheets, with a diameter of about 10 μm.^22^ The CoAl-LDH nanosheets are uniformly distributed on the surface of Ni-MOF-74 (Figure 2c,f), indicating the successful preparation of the 20-NiL composite and the effective mitigation of CoAl-LDH agglomeration. The high-resolution TEM (HRTEM) image in Figure 2g shows a distinct interface between Ni-MOF-74 and CoAl-LDH, confirming the successful formation of a composite heterojunction. The lattice fringes corresponding to the (101) plane of Ni-MOF-74 and the (012) plane of CoAl-LDH were measured to be 0.57 and 0.26 nm, respectively.^18^ Furthermore, the EDS mapping results (Figure 2h–m) clearly demonstrate the homogeneous distribution of C (purple), O (light green), Co (blue), Al (green), and Ni (cyan) elements on the surface of the composite material. Combined with the elemental content data provided in Table S2 (Supporting Information), these figures collectively provide further confirmation for the successful preparation of the 20-NiL composite catalyst.

**Figure 2:**
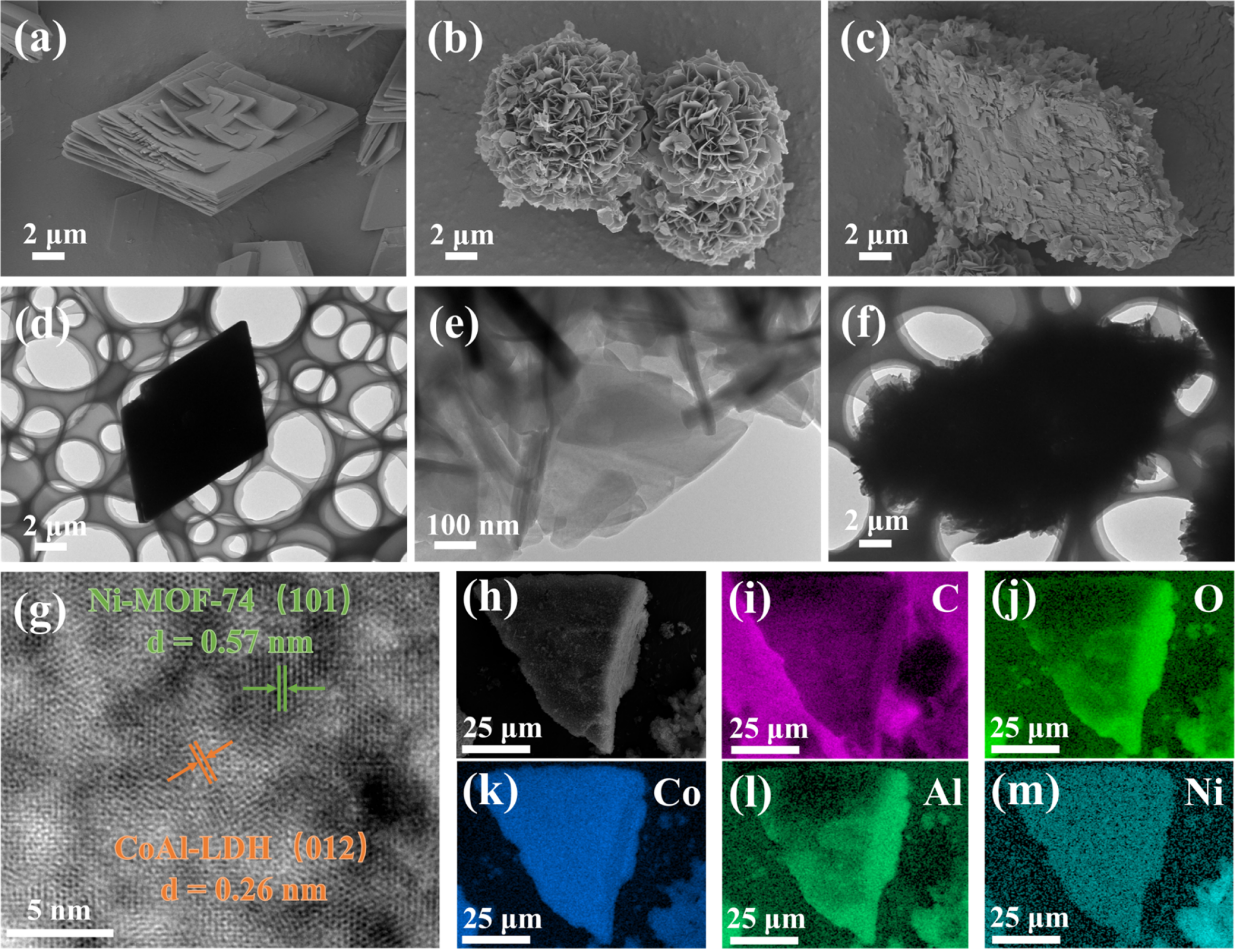
(a–c) SEM images of (a) Ni-MOF-74, (b) CoAl-LDH, and (c) 20-NiL. (d–f) TEM images of (d) Ni-MOF-74, (e) CoAl-LDH, and (f) 20-NiL. (g) HRTEM image of 20-NiL. (h–m) Corresponding EDS elemental mapping images of 20-NiL for (i) C, (j) O, (k) Co, (l) Al, and (m) Ni.

The elemental composition and chemical states of the prepared samples were investigated using X-ray photoelectron spectroscopy (XPS), with the survey spectra of all elements present in the samples shown in Figure 3. All binding energies in the XPS spectra were calibrated against the C 1s peak of adventitious carbon at 284.8 eV. The presence of signals corresponding to Co, Al, Ni, C, and O elements in the survey spectrum of the 20-NiL composite catalyst provides further evidence for its successful preparation (Figure S1). High-resolution XPS spectra were acquired to determine the valence states of each element. As shown in Figure 3a, for pristine CoAl-LDH, the peaks located at 781.28 and 797.58 eV are assigned to 2p_3/2_ and 2p_1/2_ orbitals of Co^3+^, respectively, while the peaks at 784.08 and 801.58 eV are attributed to 2p_3/2_ and 2p_1/2_ orbitals of Co^2+^.^23^ In the composite 20-NiL catalyst, the 2p_3/2_ and 2p_1/2_ orbitals for Co^3+^ are observed at 781.18 and 797.48 eV, and the 2p_3/2_ and 2p_1/2_ orbitals for Co^2+^ are found at 783.68 and 799.58 eV. The satellite peaks for both Co^2+^ and Co^3+^ are typically observed around 787 and 804 eV.^24^ As presented in Figure 3b, the Al 2p spectra of both pristine CoAl-LDH and the 20-NiL composite exhibit a single main peak at 74.48 and 74.28 eV, respectively, which is characteristic of Al^3+^. Based on these results, the introduction of Ni-MOF-74 induces a slight shift of the Co 2p and Al 2p binding energies in the 20-NiL composite toward lower values compared to pristine CoAl-LDH. This negative shift suggests an increase in electron density on the CoAl-LDH within the composite material. As shown in Figure 3c, the peaks observed at 856.38 and 874.08 eV for pristine Ni-MOF-74 are assigned to the 2p_3/2_ and 2p_1/2_ orbitals of Ni elements, respectively. Similarly, the peaks detected at 856.68 and 874.18 eV for the composite 20-NiL catalyst are also attributed to the 2p_3/2_ and 2p_1/2_ orbitals of Ni ions. The satellite peaks located around 862 and 880 eV are commonly ascribed to the oxidized state of nickel species exposed to air.^11^ A slight positive shift in the Ni 2p binding energies of the 20-NiL composite was observed compared to that of pristine Ni-MOF-74. This shift indicates a decrease in electron density on the Ni-MOF-74 component within the composite. These shift trends clearly reveal the charge redistribution at the interface following successful formation of the heterojunction: electrons transfer from Ni-MOF-74 to CoAl-LDH, resulting in a partial positive charge on the Ni-MOF-74 side due to electron loss and a partial negative charge on the CoAl-LDH side due to electron gain. This “positive–negative” charge pair establishes a built-in electric field across the interface, oriented from Ni-MOF-74 toward CoAl-LDH. Furthermore, the high-resolution O 1s and C 1s spectra of the pristine and composite samples are provided in Figure S1 (Supporting Information). In the O 1s spectrum of CoAl-LDH, deconvolution reveals three peaks at 531.68, 532.68, and 530.58 eV, which are attributed to lattice oxygen, adsorbed oxygen,^25^ and unsaturated oxygen coordination, respectively. The peak fitting results for the O 1s spectrum of 20-NiL are largely consistent with those of CoAl-LDH. In contrast, the O 1s spectrum of Ni-MOF-74 exhibits only two peaks at 531.78 and 533.28 eV, corresponding to lattice oxygen and adsorbed oxygen, respectively. For the C 1s spectra of all prepared catalysts, the dominant peak appears at 284.8 eV is due to the presence of C–C bonds.^26^ The peak observed near 286 eV is assigned to C–O bonds, while the peak around 288 eV is attributed to C=O bonds.^27^

**Figure 3:**
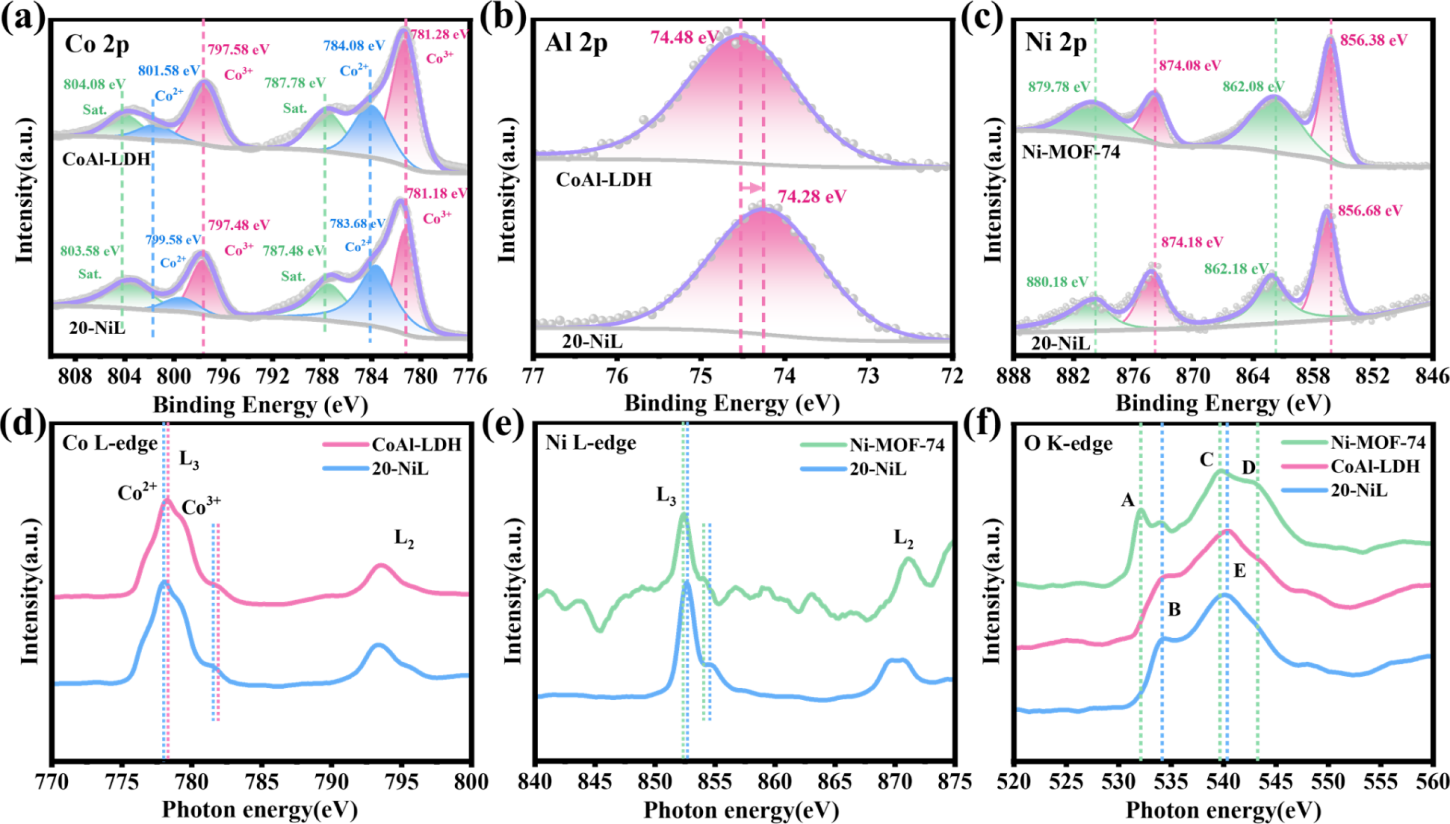
High-resolution XPS spectra of (a) Co 2p, (b) Al 2p, and (c) Ni 2p for the prepared samples before and after compositing. Soft X-ray absorption spectra of the (d) Co *L*-edge, (e) Ni *L*-edge, and (f) O *K*-edge.

Soft X-ray absorption spectroscopy (sXAS) is element-specific and highly sensitive to the electronic structure of transition metal (TM) elements. Its energy positions and spectral intensity can be utilized to probe TM valence states, spin states, coordination environments, and orbital hybridization.^28^ For TM elements, the *L*-edge absorption corresponds to dipole-allowed transitions from the 2p core level to unoccupied 3d orbitals. Due to spin–orbit coupling of the 2p orbital, the *L*-edge splits into two characteristic edges: the *L*_3_-edge (2p_3/2_ → 3d) at the lower energy side and the *L*_2_-edge (2p_1/2_ → 3d) at the higher energy side. As shown in Figure 3d, the Co *L*_2,3_ sXAS spectrum consists of two regions: the *L*_3_-edge feature near 778 eV and the *L*_2_-edge feature near 793 eV. The splitting of the Co *L*_3_-edge is related to the transition energies associated with their coordination geometry, indicating a higher spin state for Co^2+^ compared to Co^3+^.^29^ We observe that the centroid of the Co *L*_3_-edge feature in the composite 20-NiL shifts by approximately 0.3 eV toward lower photon energy compared to that in pristine CoAl-LDH, suggesting a reduction in the valence state of Co ions. This indicates that charge transfer (electron reception by CoAl-LDH) occurs at the interface following the construction of the composite heterojunction. Concurrently, the increased intensity of the Co^2+^ peak within the Co *L*_3_-edge of the composite also suggests an increase in Co^2+^ content. Combined with the XPS results, this corroborates the migration of charge from Ni-MOF-74 to CoAl-LDH. As shown in Figure 3e, the Ni *L*_3_-edge exhibits a very sharp main peak near 852.5 eV and a satellite peak (shoulder) near 854.5 eV, while the Ni *L*_2_-edge feature is observed near 870 eV, which is similar to the standard spectral features of high-spin Ni^2+^.^30^ The Ni *L*_3_-edge peak position in the composite 20-NiL shifts by approximately 0.3 eV toward higher energy compared to that in pristine Ni-MOF-74, indicating an increased valence state of Ni after compositing. This suggests that charge transfer (electron loss from Ni-MOF-74) occurs at the interface upon heterojunction formation. For the O element, the *K*-edge sXAS represents dipole-allowed transitions of electrons from the 1s orbital to unoccupied 2p orbitals. As shown in Figure 3f, pristine Ni-MOF-74 displays a distinct pre-edge feature (Peak A) near 532 eV, attributed to the hybridization of O 2p and Ni 3d orbitals. A series of broader peaks (Peaks C, D) over a wider energy range near 539 eV correspond to transitions of O 1s electrons to unoccupied states formed by the hybridization of O 2p orbitals with higher-energy orbitals such as Ni 4sp. In contrast, both pristine CoAl-LDH and the composite 20-NiL show a broad peak, where Peak B is primarily attributed to the hybridization of O 2p and Co 3d orbitals, and Peak E is mainly due to hybridization with higher-energy orbitals like Co 4sp.^31^ Furthermore, the overall intensity of the O *K*-edge features is higher in the composite sample, indicating a greater degree of hybridization and a higher density of unoccupied states, which enhances the probability of electron transitions and facilitates the separation of photogenerated charge carriers. Under dark conditions, sXAS and XPS measurements reveal consistent electron-state changes—electron depletion from Ni-MOF-74 accompanied by electron accumulation on CoAl-LDH. This observation not only directly confirms spontaneous electron transfer upon interfacial contact, but more importantly, indicates that even before light irradiation, the interface is pre-configured with a built-in electric field oriented from Ni-MOF-74 toward CoAl-LDH. This field favors a *Z*-scheme charge-separation pathway over the conventional type-II mechanism. The direction of this field predetermines that, upon photoexcitation, the most probable carrier-separation route involves recombination of photogenerated electrons from CoAl-LDH with photogenerated holes from Ni-MOF-74 at the interface. As a result, electrons in the conduction band of Ni-MOF-74 and holes in the valence band of CoAl-LDH are effectively preserved and spatially separated—exactly the defining characteristic of a direct *Z*-scheme heterojunction.

### 3.2 Photoelectrochemical Properties

The light absorption properties and band gap information (*E*_g_) of the catalytic materials were determined using ultraviolet–visible diffuse reflectance spectroscopy (UV–vis DRS), as shown in Figure 4a. Both the pristine CoAl-LDH and the composite heterojunction catalysts exhibit distinct absorption valleys in the ultraviolet region near 250–300 nm, which are commonly attributed to ligand (O^2–^) to metal (Co^2+^) charge transfer (LMCT). The d–d orbital transitions of octahedral Co^2+^ within the CoAl-LDH layers occur at approximately 450, 490, and 530 nm in the visible region. The absorption peak intensity at around 630 nm, corresponding to the d–d transition of octahedrally coordinated low-spin Co^3+^, is significantly lower than that of Co^2+^. Notably, the pristine Ni-MOF-74 displays two prominent absorption bands at 300–350 nm and 400–500 nm. The former is assigned to the π–π* electronic transition of the ligand itself, while the latter is ascribed to ligand-to-metal charge transfer (LMCT) transition. The LMCT process is crucial for the visible-light response of pure Ni-MOF-74, enabling photogenerated electrons to be effectively localized at the Ni^2+^ active sites while holes are delocalized over the organic ligand framework.^32^ This achieves a preliminary spatial separation of photogenerated charges within the material, thereby laying an electronic structural foundation for constructing a *Z*-scheme heterojunction with CoAl-LDH.

**Figure 4:**
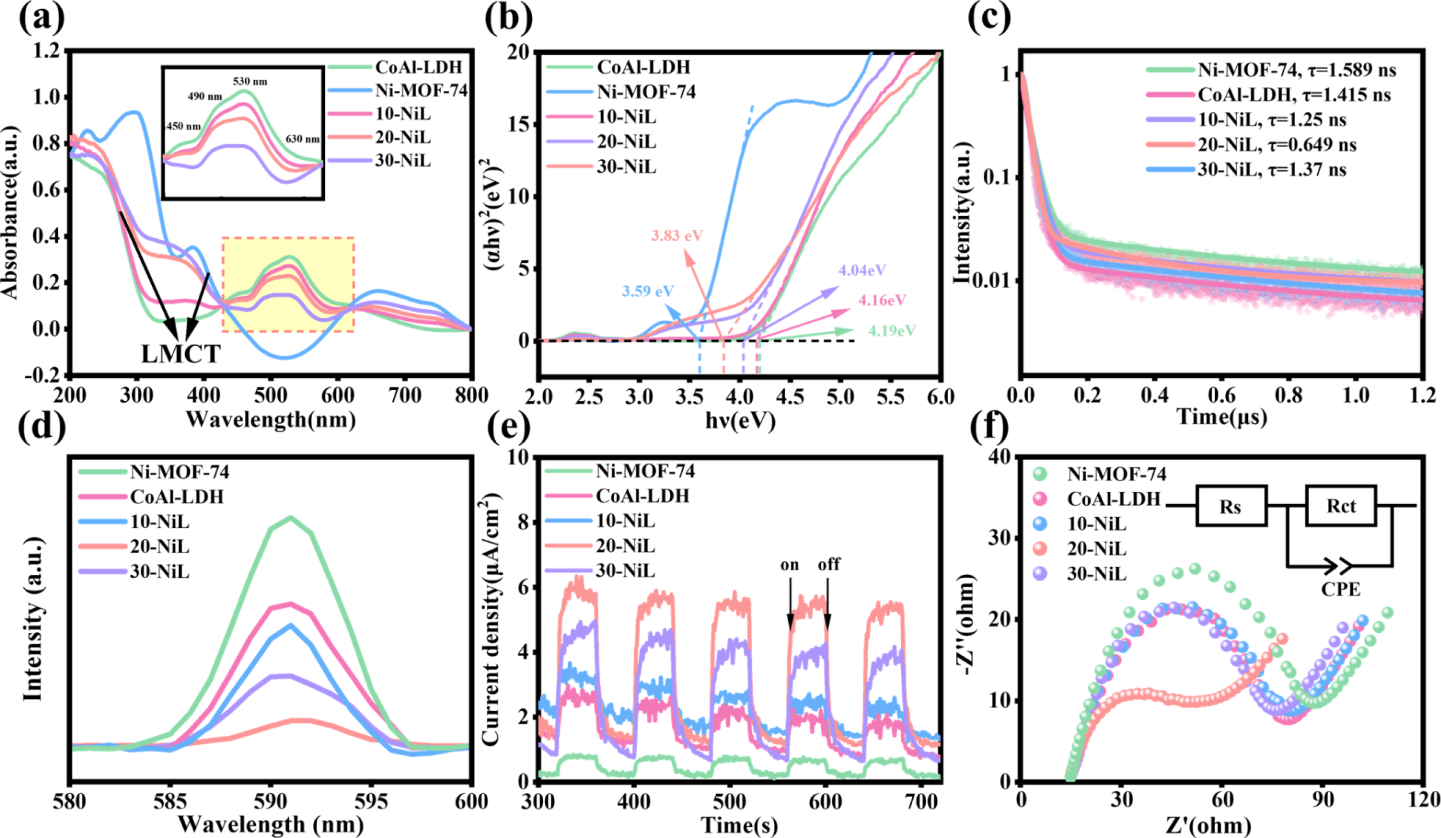
(a) UV–vis diffuse reflectance spectra (UV–vis DRS). (b) the corresponding Tauc plots. (c) time-resolved photoluminescence (TRPL) spectra. (d) photoluminescence (PL) spectra. (e) transient photocurrent responses, and (f) electrochemical impedance spectroscopy (EIS) Nyquist plots of the as-prepared samples.

The band gap (*E*_g_) of pristine CoAl-LDH was calculated to be approximately 4.19 eV by constructing a Tauc plot (Figure 4b) based on the Kubelka–Munk function.^33^ Similarly, the band gap of pristine Ni-MOF-74 was estimated to be about 3.59 eV from the corresponding Tauc plot. The band gaps of 10-NiL, 20-NiL, and 30-NiL were calculated to be 4.16, 4.04, and 3.83 eV, respectively. The progressively narrowing band gap indicates a corresponding enhancement in their light-harvesting capability under simulated solar illumination. When excited at a wavelength of 485 nm, the composite catalyst 20-NiL demonstrates the lowest photoluminescence (PL) intensity compared to Ni-MOF-74 and CoAl-LDH (Figure 4d). This indicates the highest separation efficiency of electron–hole pairs (e^–^–h^+^) and the most effective suppression of photogenerated charge carrier recombination within 20-NiL.^34^ The time-resolved photoluminescence (TRPL) spectra of all samples, obtained under excitation at 480 nm, are presented in Figure 4c. The fitting results of the fluorescence lifetime decay curves reveal that among all tested samples, Ni-MOF-74 possesses the longest average fluorescence lifetime (τ = 1.589 ns), whereas 20-NiL exhibits the shortest (τ = 0.649 ns). The average fluorescence lifetime typically reflects the competition between radiative and nonradiative recombination through the survival time of photogenerated charge carriers. Ideally, nonradiative recombination pathways dominate the catalytic reaction. In this case, an extremely short fluorescence lifetime indicates rapid charge transfer and highly efficient charge separation.^35^ This signifies that the charge carriers are not lost through fluorescent recombination but are instead rapidly injected into the conduction or valence bands and captured for catalytic reactions. This finding demonstrates that the formation of a heterostructure between Ni-MOF-74 and CoAl-LDH effectively accelerates the charge migration rate of photogenerated carriers, thereby enhancing the transfer and utilization efficiency of activated electrons in the PCO_2_RR. The transient photocurrent response was measured over five cycles with the light source switched on and off at 40 s intervals (Figure 4e). A higher steady-state photocurrent density indicates a stronger promoting effect of the heterojunction on charge carrier separation.^36^ The composite catalyst 20-NiL exhibited a significantly higher photocurrent density compared to the other samples, indicating the highest electron yield and charge carrier separation efficiency in the PCO_2_RR. As shown in Figure 4f, **Rs** represents the resistance of the electrolyte solution between the working electrode and the reference electrode in the equivalent circuit. Since the charge transfer resistance (**Rct**) at the heterojunction interface often changes upon composite formation, it corresponds to the variation in the diameter of the semicircle in the Nyquist plot. Comparing all tested catalysts, the composite catalyst 20-NiL exhibits the smallest arc radius, indicating the lowest interfacial impedance and the highest conductivity.^37^ Collectively, these results demonstrate that the constructed composite catalyst *x*-NiL effectively facilitates the separation and transfer of photogenerated charge carriers, suppresses the recombination of electron–hole (e^–^–h^+^) pairs during the catalytic process, and consequently enhances the photocatalytic activity and efficiency.

### 3.3 Photocatalytic CO_2_ Reduction Performance

Since pristine CoAl-LDH typically exhibits low photocatalytic activity in the absence of sacrificial agents and photosensitizers, the photocatalytic reduction tests in this work were conducted in a liquid–solid reaction system employing triethanolamine (TEOA) as the sacrificial agent for holes (h^+^), Ru­(bpy)_3_Cl_2_·6H_2_O as the photosensitizer, and a xenon lamp (λ = 320–780 nm) as the light source.^38^ As shown in Figure 5a and calculated using Equation S2 (Supporting Information), the main products after 1 h of illumination in the system containing the sacrificial agent and photosensitizer were CO, CH_4_, and H_2_ (Figure S2, Supporting Information). The CO production rates for pristine CoAl-LDH and pristine Ni-MOF-74 were 47.04 and 47.60 μmol·g^–1^·h^–1^, respectively. The composite catalysts 10-NiL, 20-NiL, and 30-NiL exhibited CO production rates of 62.20, 79.86, and 56.63 μmol·g^–1^·h^–1^, respectively. A significant enhancement in the CO production rate is observed for the composite samples compared to the pristine catalysts, with 20-NiL showing the highest rate, representing an improvement of nearly 70% over both pristine CoAl-LDH and Ni-MOF-74. Concurrently, the H_2_ production rates for CoAl-LDH, 10-NiL, 20-NiL, 30-NiL, and Ni-MOF-74 were 141.45, 201.82, 290.57, 212.03, and 61.10 μmol·g^–1^·h^–1^, respectively. The variation trend in H_2_ production is largely consistent with that of CO production. In addition to CO and H_2_, trace amounts of CH_4_ were detected, with production rates of 0.109, 0.084, 0.133, 0.091, and 0.202 μmol·g^–1^·h^–1^ for CoAl-LDH, 10-NiL, 20-NiL, 30-NiL, and Ni-MOF-74, respectively. The gradual enhancement in photocatalytic activity with the introduction of Ni-MOF-74 is primarily attributed to the formation of the *Z*-scheme heterojunction, which promote CO_2_ activation and the reduction of H_2_O (H^+^ → H_2_). However, when the mass ratio of Ni-MOF-74 increased from 20% to 30%, the photocatalytic activity began to decline. This suggests that an excessive amount of Ni-MOF-74 may shield the active sites for photocatalytic reduction, thereby limiting the mass transfer of reactants (CO_2_/H_2_O).^39^ As shown in Figure 5b, 20-NiL maintained relatively stable photocatalytic reduction activity even after six consecutive cycling tests, with the photocatalytic performance in the sixth cycle retaining 91% of the initial activity. The SEM images and XRD patterns of the composite sample after multiple cycles (Figures S3–S4, Supporting Information) indicate mild agglomeration; however, the XRD patterns before and after the reaction show no significant changes in the structure and crystal phase of the composite catalyst. This confirms the good stability and low degree of photocurrosion of the catalyst during the reaction process. To investigate the roles of TEOA and the photosensitizer and to unequivocally confirm that the carbon source of the products originates solely from CO_2_ reduction, control experiments under different reaction conditions (Figure 5c) and isotopic labeling experiments (Figure 5d) were performed. The control experiments were conducted under an Ar atmosphere, in the dark, without the catalyst, and without TEOA and the photosensitizer. The results confirm that the products are generated only when the catalyst is present under illumination in a CO_2_ atmosphere containing the sacrificial agent and photosensitizer. For the isotopic labeling experiments, ^12^CO_2_ and ^13^CO_2_ were used separately as the sole carbon source under identical reaction conditions. The resulting mass spectra showed distinct peaks for ^12^CH_4_ (*m*/*z* = 16) and ^13^CH_4_ (*m*/*z* = 17), as well as for ^12^CO (*m*/*z* = 28) and ^13^CO (*m*/*z* = 29). The isotopic analysis, combined with the control experiments, unequivocally demonstrates that the CO and CH_4_ products originate solely from the PCO_2_RR.^40^ Furthermore, to objectively evaluate the PCO_2_RR performance of 20-NiL in this work, we have compared it with other recently reported photocatalysts of a similar class (Table S3, Supporting Information). The comparison clearly demonstrates that 20-NiL achieves superior catalytic efficiency for CO_2_-to-CO conversion.

**Figure 5:**
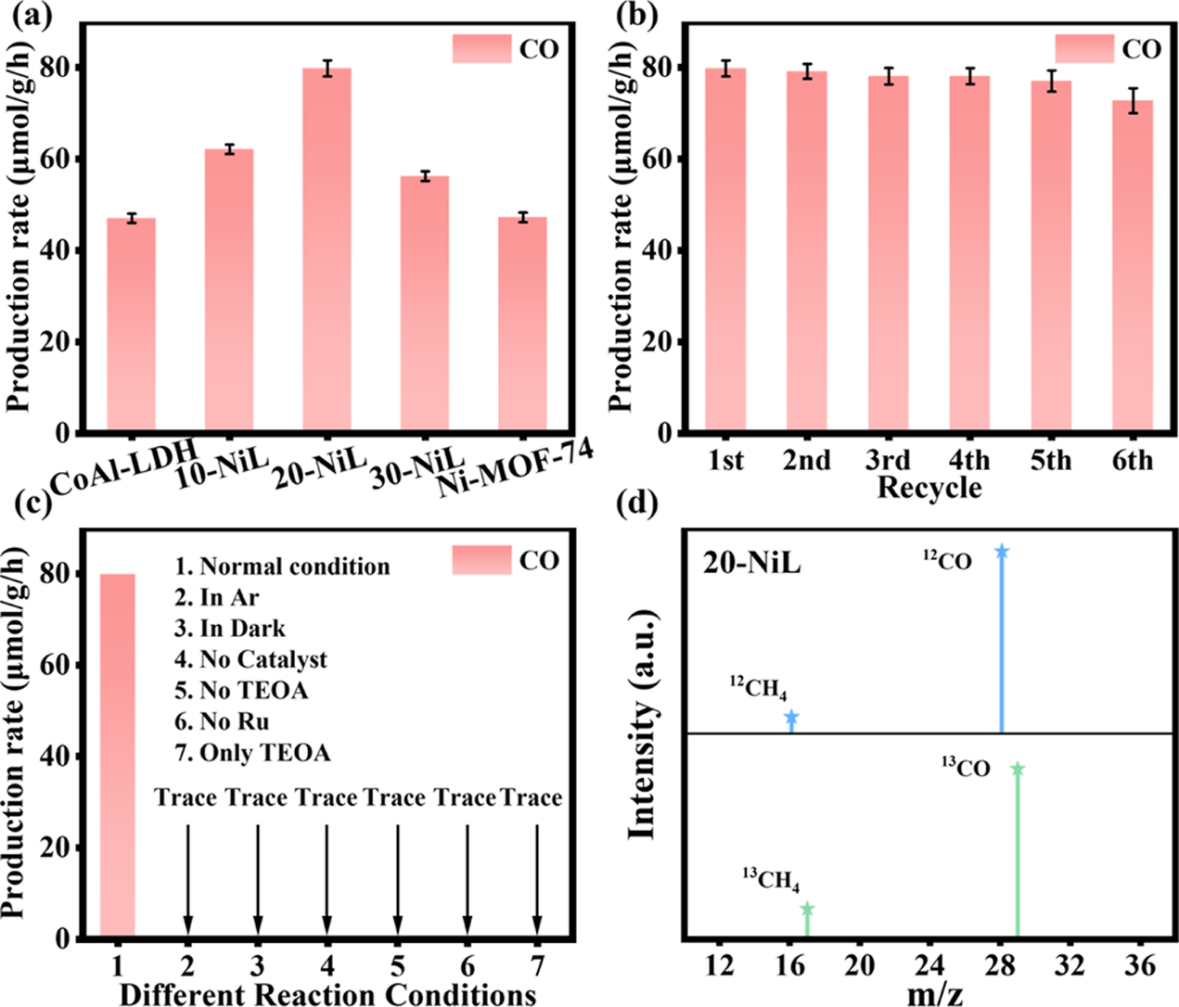
(a) CO production rate of different photocatalysts. (b) CO production rate of 20-NiL during cycling tests. (Each data was measured three times independently to obtain the average value and the standard deviation) (c) CO production rates under different reaction conditions. (d) Mass spectra of the photocatalytic products generated over 20-NiL under ^12^CO_2_ and ^13^CO_2_ atmospheres.

### 3.4 Analysis of the Photocatalytic Mechanism

In situ Fourier Transform Infrared Spectroscopy (in situ FTIR) was employed to gain deeper insights into the photocatalytic CO_2_ reduction process and monitor the dynamic evolution of intermediate species. An image of the experimental equipment used for these measurements is provided in Figure S5. The spectral results shown in Figure 6 reveal characteristic peaks for monodentate carbonate (m-CO_3_^2–^) at approximately 1506, 1501, and 1498 cm^–1^ for pristine CoAl-LDH, the 20-NiL composite and pristine Ni-MOF-74, respectively. Peaks corresponding to bidentate carbonate (b-CO_3_^2–^) were observed around 1366, 1361, and 1436 cm^–1^, while bicarbonate ion (b-HCO_3_^–^) exhibits peaks at approximately 1715, 1731, and 1733 cm^–1^.^41–45^ These observations indicate the adsorption and activation of CO_2_ and H_2_O molecules on the catalyst surfaces. The crucial intermediate COOH* exhibits peaks at approximately 1630 and 1280 cm^–1^, whose signal intensities increase with prolonged illumination time.^3,46^ Notably, absorption peaks for *CHO were observed at approximately 1102 cm^–1^, and the intensities of the peaks for *CH_3_O at around 1016 and 1148 cm^–1^ also intensified over time.^47^ This confirms that the presence of Ni-MOF-74 facilitates the generation of more electrons, which assist adsorbed *COOH species in accepting electrons to form *CHO and *CH_3_O intermediates, recognized as essential precursors for CH_4_ formation.^48^ Finally, peaks corresponding to the final intermediate *CO and its desorbed product CO (g) were detected near 2077 and 2185 cm^–1^, respectively.^49^ Based on these findings, a potential reaction mechanism for the PCO_2_RR in this study can be proposed as follows:

1 $$CO_{2}\left(g\right)+*\to CO_{2}^{*}$$

2 $$CO_{2}^{*}+H^{+}+e^{-}\to {COOH}^{*}$$

3 $${COOH}^{*}+H^{+}+e^{-}\to {CO}^{*}{+H}_{2}O$$

4 $${CO}^{*}\to CO\left(g\right)$$

5 $$orCO_{2}\left(g\right)+*\to CO_{2}^{*}$$

6 $$CO_{2}^{*}+H^{+}+e^{-}\to {COOH}^{*}$$

7 $${COOH}^{*}+H^{+}+e^{-}\to {CO}^{*}{+H}_{2}O$$

8 $${CO}^{*}+H^{+}+e^{-}\to {CHO}^{*}$$

9 $${CHO}^{*}+H^{+}+e^{-}\to {CH_{2}O}^{*}$$

10 $${CH_{2}O}^{*}+H^{+}+e^{-}\to {CH_{3}O}^{*}$$

11 $${CH_{3}O}^{*}+H^{+}+e^{-}\to CH_{4}\left(g\right)+O^{*}$$

**Figure 6:**
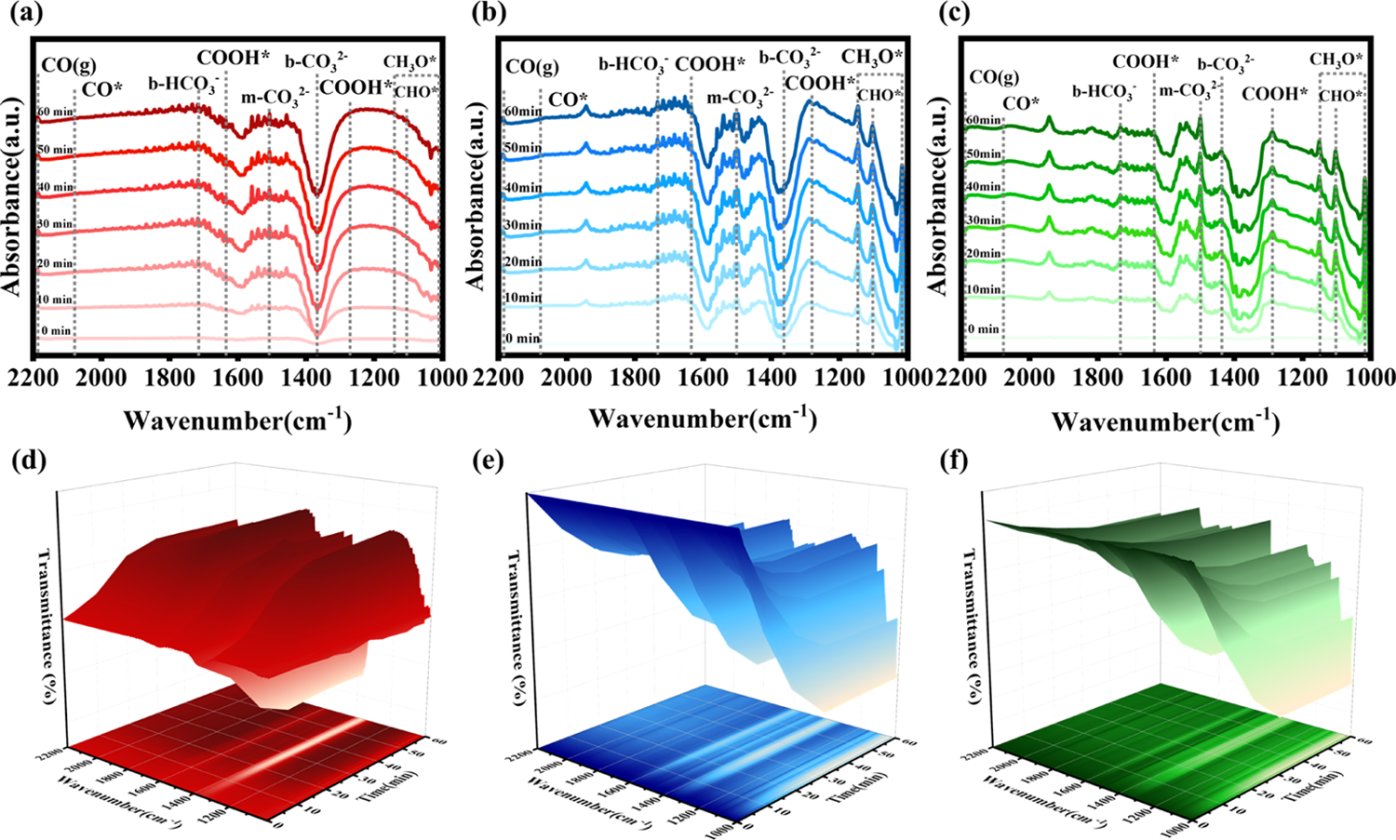
In situ FTIR spectra of (a) CoAl-LDH (red curve), (b) 20-NiL (blue curve), and (c) Ni-MOF-74 (green curve). The corresponding 3D color-mapped surface plots with projections for (d) CoAl-LDH, (e) 20-NiL, and (f) Ni-MOF-74.

To further elucidate the charge transfer mechanism, high-resolution XPS analysis was conducted on the composite material after photocatalysis. As shown in Figure 7a–c, the binding energy of the Co^3+^ 2p_3/2_ peak increased from 781.08 to 781.38 eV, while that of the Co^3+^ 2p_1/2_ peak increased from 797.48 to 797.58 eV. Concurrently, the binding energy of the Co^2+^ 2p_3/2_ peak was observed to shift from 783.68 to 784.28 eV, and that of the Co^2+^ 2p_1/2_ peak from 799.58 to 801.08 eV. Notably, the significant reduction in the spectral weight of the Co^2+^ 2p_3/2_ peak indicates electron loss from the cobalt centers, providing key evidence for the oxidation of cobalt (i.e., an increase in its valence state). Similarly, the binding energy of Al 2p also exhibited an upward shift, from 74.28 to 74.53 eV. Concurrently, the Ni 2p peaks exhibited a shift toward lower binding energies. These observations indicate that after illumination, electrons (e^–^) transferred from CoAl-LDH to Ni-MOF-74 within the composite. The Mott–Schottky curves of Ni-MOF-74 and CoAl-LDH were measured at frequencies of 500, 1000, and 1500 Hz using a three-electrode system.^50^ As shown in Figure 7d, the positive slopes of the Mott–Schottky plots for both materials confirm their *n*-type semiconductor characteristics.^51^ The flat-band potentials (*E*_fb_) relative to the Ag/AgCl electrode were determined from the *x*-intercepts of the tangent lines to the Mott–Schottky curves, yielding values of −1.03 eV for Ni-MOF-74 and −0.82 eV for CoAl-LDH. These values were converted to the normal hydrogen electrode (NHE) scale using the equation *E*_NHE_ = *E*_Ag/AgCl_ + 0.2 eV, resulting in *E*_fb_ values of −0.83 and −0.62 eV versus NHE, respectively. For *n*-type semiconductors, the conduction band potential (*E*_CB_) is typically considered to be 0.1 eV more negative than the *E*_fb_. Therefore, the *E*_CB_ values of Ni-MOF-74 and CoAl-LDH were calculated to be −0.93 and −0.72 eV versus NHE, respectively.^52,53^ These *E*_CB_ values are sufficiently negative to drive the reduction of CO_2_ to CO or CH_4_. The valence band potentials (*E*_VB_) were calculated using the equation *E*_VB_ = *E*_CB_ + *E*_g_,^54^ yielding values of 2.66 eV for Ni-MOF-74 and 3.47 eV for CoAl-LDH versus NHE. As shown in Figure 7e, the XPS valence band (XPS-VB) spectra reveal that the valence band maximum (VBM) values for CoAl-LDH and Ni-MOF-74 are located at 1.96 and 1.35 eV, respectively. Using the equation– φ – *E*_VB‑XPS_ = *E*_VB(Vacuum)_,^55^ where φ is the work function of the spectrometer, the VBM values relative to the vacuum level were calculated to be −6.16 eV for CoAl-LDH and −5.55 eV for Ni-MOF-74. Combining these with the band gap information, the conduction band minimum (CBM) values relative to the vacuum level were determined to be −1.97 eV for CoAl-LDH and −1.96 eV for Ni-MOF-74. Based on the characterization results from UV–vis DRS, Mott–Schottky, and XPS-VB analyses, the band alignment of the 20-NiL composite heterojunction relative to the standard hydrogen electrode and vacuum level was constructed (Figure 7f).

**Figure 7:**
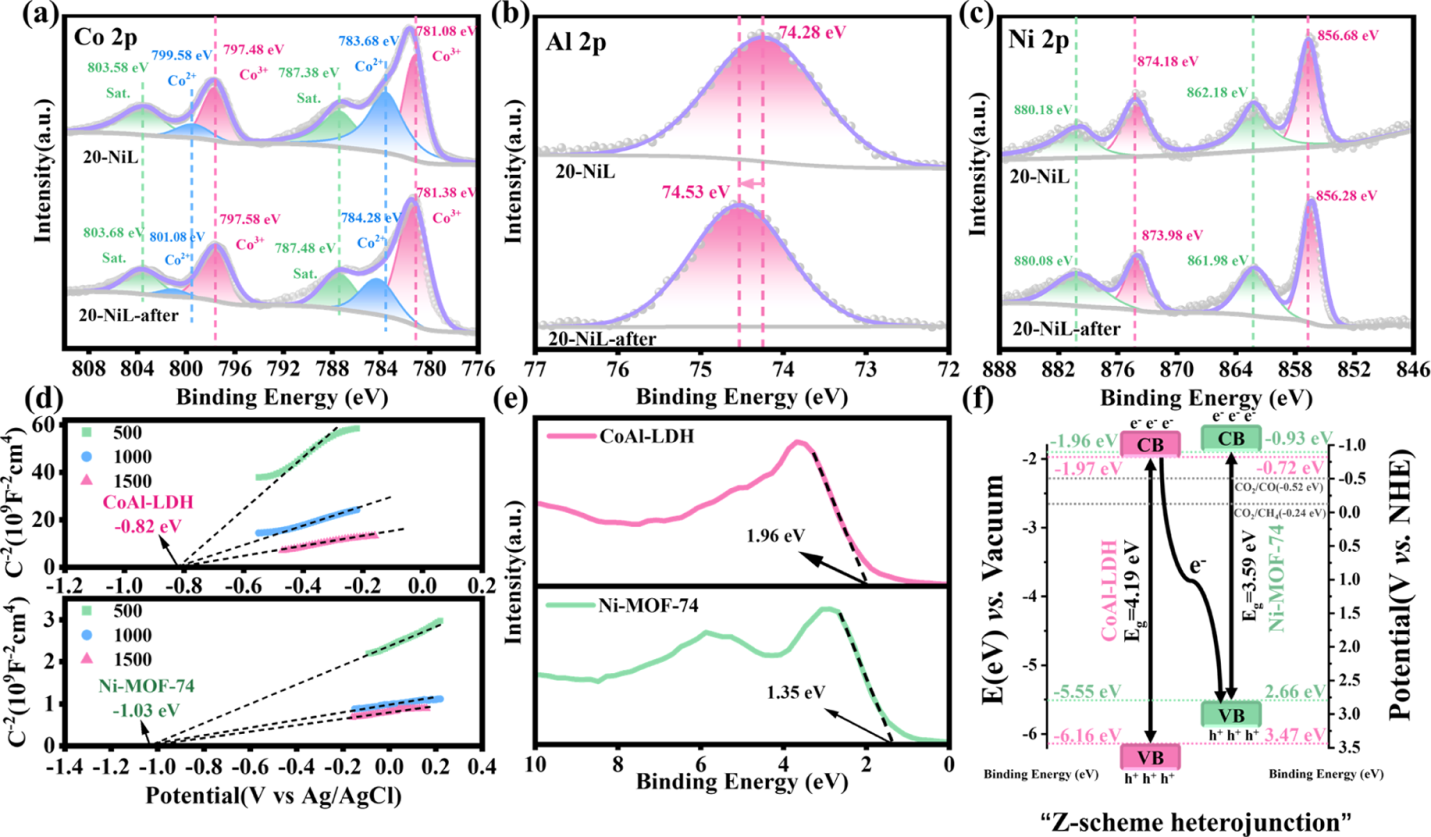
(a–c) High-resolution XPS spectra of (a) Co 2p, (b) Al 2p, and (c) Ni 2p for the composite sample before and after light irradiation. (d) Mott–Schottky plots of Ni-MOF-74 and CoAl-LDH. (e) XPS valence band (XPS-VB) spectra. (f) schematic illustration of the proposed reaction mechanism.

Based on the characterization results presented above, the charge-transfer mechanism of the constructed *Z*-scheme composite heterojunction 20-NiL is clarified in this work. Under dark conditions, directional electron flow occurs at the interface upon material hybridization, where Ni-MOF-74 loses electrons and becomes positively charged while CoAl-LDH gains electrons and becomes negatively charged, leading to the formation of an internal electric field directed from Ni-MOF-74 toward CoAl-LDH. The presence of the internal electric field confirms the occurrence of band bending at the interface (Figure S7). Under photoexcitation, photogenerated electron–hole pairs are separated. The photogenerated electrons in the conduction band of CoAl-LDH recombine at the interface with the photogenerated holes in the valence band of Ni-MOF-74, while the holes left behind in the valence band of CoAl-LDH oxidize TEOA. Simultaneously, a large number of electrons accumulate in the conduction band of Ni-MOF-74 and are delivered to the Ni active sites. These electrons directly participate in CO_2_ activation, causing bending of the CO_2_ molecule and weakening of the C=O bonds. Subsequently, a conduction-band electron and a proton (H^+^) act cooperatively to attack the activated CO_2_ molecule, thereby generating the key COOH intermediate. The higher intensity of the COOH* signal observed in situ FTIR for the heterojunction compared to the individual materials serves as the most direct evidence that electrons in the conduction band of Ni-MOF-74 are specifically concentrated and efficiently utilized by the *Z*-scheme architecture. On the other hand, the sufficiently negative conduction band potential confirms its thermodynamic capability to overcome the CO_2_/CO reduction potential (−0.52 eV vs NHE). The photosensitizer in the system (Ru­(bpy)_3_Cl_2_·6H_2_O) not only enhances light-harvesting efficiency, but also is excited to form the [Ru­(bpy)_3_]^+^ species, supplying a substantial number of electrons to the photocatalyst under illumination.

## 4 Conclusion

In summary, a direct *Z*-scheme heterojunction denoted as *x*-NiL was successfully prepared via a hydrothermal method, in which ultrathin CoAl-LDH nanosheets were constructed using Ni-MOF-74 as a structural template. The MOF template effectively suppressed the aggregation of LDH, resulting in a 63% increase in the specific surface area of the optimal sample (20-NiL) to 35.5 m^2^·g^–1^. Under dark conditions, sXAS and XPS analyses directly confirmed the formation of a built-in electric field at the interface due to spontaneous electron transfer. This significantly lowered the energy barrier for photogenerated-electron transfer and reduced the fluorescence lifetime to 0.649 ns. Meanwhile, in situ DRIFTS tracked the accumulation of key reaction intermediates along the CO_2_ → COOH* → CO* → CO pathway. Combined with postreaction XPS results, these data collectively elucidate that the direct *Z*-scheme heterojunction drives the directional migration of photogenerated electrons from CoAl-LDH to the conduction band of Ni-MOF-74. This mechanism efficiently promotes the 2e^–^ reduction pathway from CO_2_ to CO, ultimately enabling 20-NiL to achieve a CO production rate of 79.86 μmol·g^–1^·h^–1^ (a 70% enhancement over the single-component materials) while maintaining 91% of its initial activity over six consecutive cycles. This work establishes a design principle based on constructing MOF-templated direct *Z*-scheme heterojunctions, providing a potential guideline for achieving highly selective photocatalytic CO_2_ reduction.

## Supplementary Material



## Notes

Detailed experimental procedures and instrumental parameters, including fundamental material characterization, photoelectrochemical measurements, and photocatalytic CO_2_ reduction measurements, are provided in the Supporting Information (PDF)
