# Supplementary material for: Constructing Z‑Scheme Ni-MOF-74/CoAl-Layered Double Hydroxide Heterojunctions for Enhanced Photocatalytic CO2 Reduction
Source: ACS Appl Mater Interfaces. 2026 Feb 17;18(8):12681–92. doi: 10.1021/acsami.5c23229 (PMC12964342; doi:10.1021/acsami.5c23229)
Supplement: Supplementary file 1 [file am5c23229_si_001.pdf]

Supporting Information

Constructing Z-Scheme Ni-MOF-74/CoAl-layered Double Hydroxide Heterojunctions for Enhanced Photocatalytic CO_2 Reduction

Can Wang[a], Zhiyao Wu[b], Mengwei Chen[a], Yuxiang Deng[a], Guilin He[a], Xinpeng Wang[a], YanQiu Zhu[a,c]*, Nannan Wang[a]*

[a] State Key Laboratory of Featured Metal Materials and Life-cycle Safety for Composite Structures, MOE Key Laboratory of New Processing Technology for Nonferrous Metals and Materials, and School of Resources, Environment and Materials, Guangxi University, Nanning 530004, China.

[b] State Key Laboratory of Chemistry for NBC Hazards Protection, Frontiers Science Center for Rare Isotopes, School of Nuclear Science and Technology, Lanzhou University, Lanzhou 730000, China.

[c] Faculty of Environment, Science and Economy, University of Exeter, EX4 4QF, United Kingdom

**Corresponding author. E-mail address: wangnannan@gxu.edu.cn (Nannan Wang)*

Text. S1 Materials characterization measurements

Material characterization was performed using scanning electron microscopy (SEM, Zeiss Sigma 300) to investigate surface morphology and microstructure at an accelerating voltage of 5 kV, coupled with energy-dispersive spectroscopy (EDS) for elemental composition and distribution analysis. High-resolution transmission electron microscopy (TEM, JEOL JEM-2100Plus) was employed to examine atomic-scale morphological features and lattice information through high-energy electron beam penetration of ultrathin specimens.

X-ray diffraction (XRD) analysis was performed on a Rigaku SmartLab diffractometer equipped with a copper anode X-ray source (λ=0.15418 nm), operating at 40 kV accelerating voltage and 150 mA tube current. Scans were conducted from 5°-85° at a rate of 8°/min to characterize the crystalline structure of the photocatalysts. Fourier transform infrared (FT-IR) spectra were acquired using a Thermo Scientific Nicolet iS50 spectrometer over the range of 4000-400 cm^{-1} to identify surface functional groups and chemical bonding configurations. Specific surface areas were determined from N_2 adsorption-desorption isotherms measured on a Micromeritics ASAP 2020 analyzer, with calculations based on the Brunauer-Emmett-Teller (BET) method.

Elemental composition and chemical states were analyzed using an X-ray Photoelectron Spectroscopy (XPS, Thermo Scientific K-Alpha) with all binding energies calibrated against the C 1s peak at 284.8 eV. Soft X-ray Absorption Spectroscopy (sXAS) were acquired at Beamline BL12B-a of the National Synchrotron Radiation Laboratory (NSRL, Hefei, China) under ultra-high vacuum conditions (5×10^{-8} Pa) at 298 K. Total electron yield (TEY) mode was employed to probe absorption characteristics in the soft X-ray range (50-2000 eV), revealing elemental valence states, spin configurations, and surface-localized electronic information. The reaction pathway and intermediates during photocatalytic CO_2 reduction were monitored through *in situ* Fourier Transform Infrared Spectroscopy (*in situ* FTIR, Nicolet iS50). The reaction cell was purged with argon for 30 minutes to eliminate atmospheric contaminants, with subsequent spectra serving as background references. The CO_2/H_2O vapor mixture (10 mL/min) was then introduced for 10 minutes adsorption.

Photoreaction was initiated using a 300W xenon lamp (PLS-SXE300D, λ=320-780 nm) with continuous spectral acquisition throughout the 50-minute illumination period. The image of the laboratory testing equipment is shown in Fig. S5.

Text. S2 Photoelectrochemical measurements

The optical band gap (E_g) of the nanocatalytic material was determined using UV-Vis Diffuse Reflectance Spectroscopy (UV-Vis-DRS, UV-3600 Plus) over the spectral range of 200-800 nm. The diffuse reflectance spectral data were first converted into absorption spectra through the Kubelka-Munk function (**Equation S1**), and the band gap was subsequently calculated using the derived Tauc plot.

$$(\alpha h\nu)^{\frac{1}{n}} = K\left(h\nu - E_g\right) \quad \text{(S1)}$$

where ν represents the frequency of light across different spectral regions, h denotes Planck's constant, while K and α are the proportionality constant and absorption coefficient, respectively. The term E_g corresponds to the band gap energy (eV). For direct band gap semiconductor materials, the exponent n is 1/2, whereas for indirect band gap semiconductors, n takes a value of 2. The abscissa of the Tauc plot is $h\nu$, the ordinate is $(\alpha h\nu)^{1/n}$, and the band gap energy corresponds to the extrapolated intercept value of the slope tangent to the linear region with the x-axis.

Photoelectrochemical tests were conducted using an electrochemical workstation (CHI-660D) to investigate the band structure and charge separation efficiency of the photocatalyst. These tests employed a three-electrode system with uniformly loaded fluorine-doped tin oxide conductive glass as the working electrode, solid-state Ag/AgCl as the reference electrode, a Pt sheet as the counter electrode, and 0.5 M sodium sulfate solution as the electrolyte. Measurements included Mott-Schottky curves at 500 Hz, 1000 Hz, and 1500 Hz, transient photocurrent response spectroscopy under visible light irradiation from a 300 W xenon lamp (λ = 320-780 nm), and electrochemical impedance spectroscopy (EIS). Photoluminescence (PL) and time-resolved photoluminescence (TRPL) spectra were measured using a high-sensitivity transient fluorescence spectrometer (FL3C-111 TCSPC) to reveal the fluorescence average lifetime and recombination pathways of photogenerated

carriers (electron-hole pairs) in the studied material.

Text. S3 Photocatalytic CO_2 reduction measurements

The photocatalytic CO_2 reduction experiment was conducted in a 30 mL glass vial under liquid-solid phase conditions. First, 10 mg of the sample and 3.3 mg of photosensitizer ($Ru(bpy)_3Cl_2 \cdot 6H_2O$) were uniformly dispersed in a mixed solution containing 6 mL acetonitrile, 2 mL triethanolamine (TEOA), and 2 mL ultrapure (UP) water. Subsequently, high-purity CO_2 gas was continuously purged through the vial at 100 mL/min for 30 minutes to exhaust potentially interfering gases. Finally, the vial was irradiated for one hour using a 300 W xenon lamp (PLS-SXE300D, λ=320-780 nm) as the visible light source. Following illumination, gaseous products were collected via a check-valve-equipped syringe and analyzed by gas chromatography (GC-2014C, equipped with FID and TCD detectors). The quantification of carbon monoxide (CO) and methane (CH_4) followed **Equation S2**:

$$Rate = \frac{A \cdot V}{22.4 \cdot M \cdot T} \text{(S2)}$$

Where *Rate* is the production rate (unit: $\mu mol \cdot g^{-1} \cdot h^{-1}$), *A* is the detected gas concentration (unit: ppm), *V* is the reaction gas volume (unit: L), *M* is the mass of photocatalyst used (unit: g), and *T* is the irradiation time (unit: h).

Under identical photoreduction conditions, the apparent quantum yield (AQY) was determined using monochromatic light irradiation at 420 nm. The incident light intensity was measured using a USR-40 spectroradiometer (Ushio Inc., Japan), and the AQY was calculated according to the following equation: AQY (%) = 100% × (number of reacted electrons) / (number of incident photons). The apparent quantum yields (AQYs) for CO production were measured for CoAl-LDH, Ni-MOF-74, and 20-NiL, yielding values of 1.12%, 1.07%, and 1.33%, respectively.

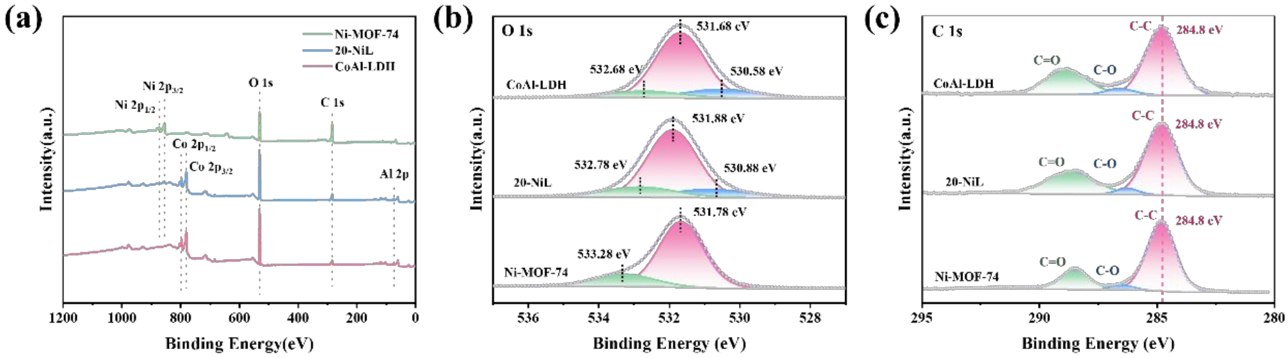

Fig. S1 (a) XPS survey spectra and (b, c) high-resolution XPS spectra of the O 1s and C 1s regions for Ni-MOF-74, CoAl-LDH and 20-NiL.

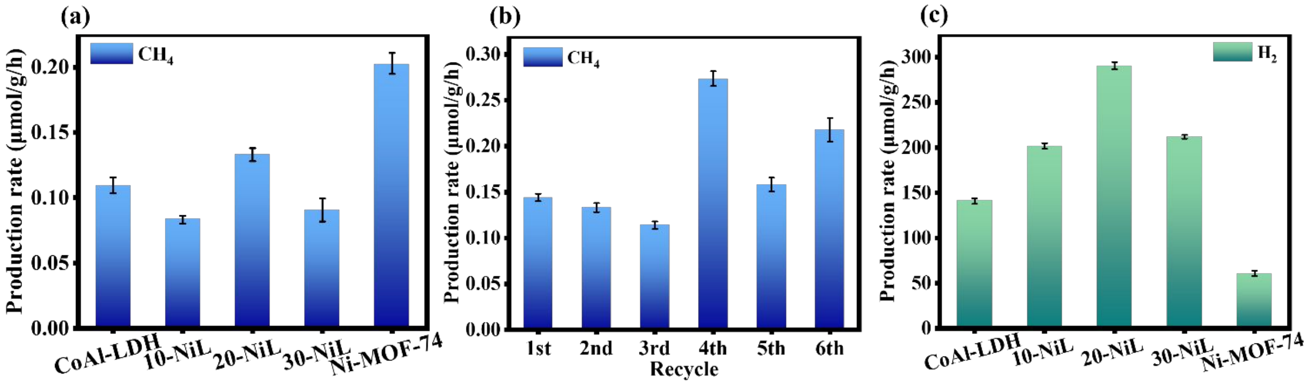

Fig. S2 (a) CH_4 production rate of different photocatalysts. (b) CH_4 production rate of 20-NiL during cycling tests. (c) H_2 production rate of different photocatalysts. (Each data was measured three times independently to obtain the average value and the standard deviation.)

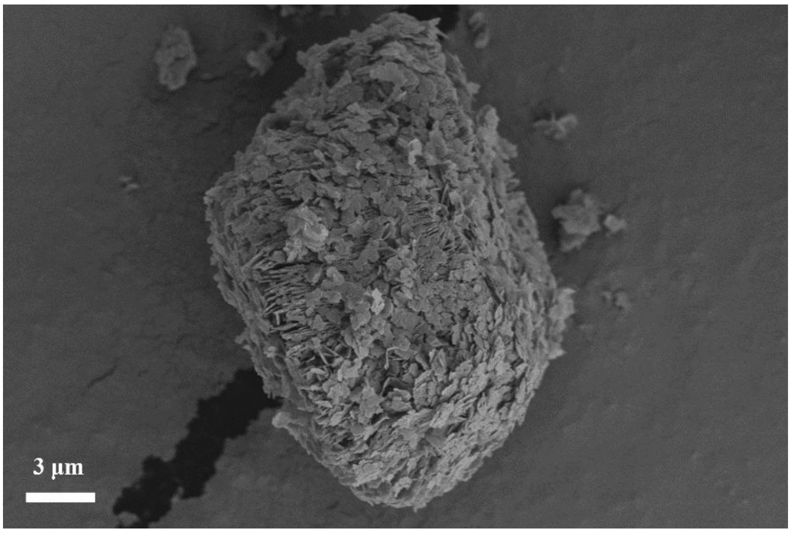

Fig. S3 SEM patterns of 20-NiL before and after 4 recycles.

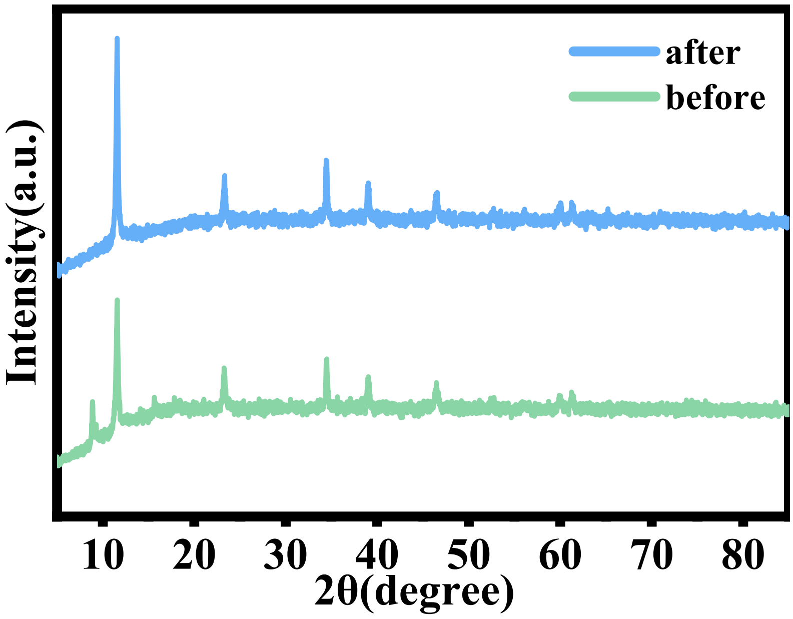

Fig. S4 XRD patterns of 20-NiL before and after 4 recycles.

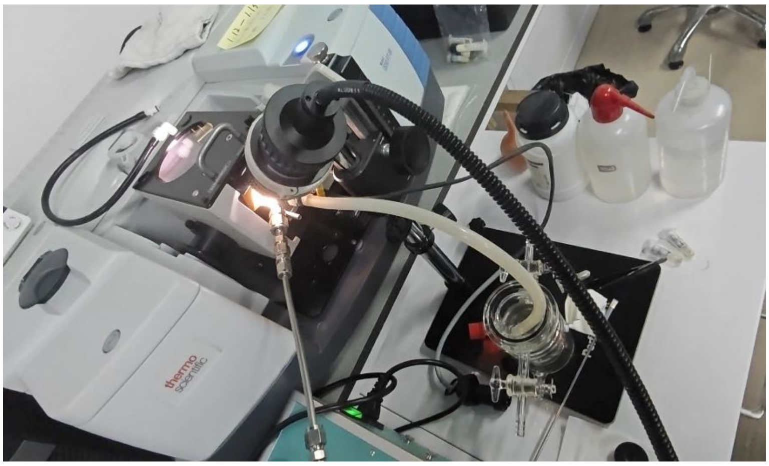

Fig. S5 The image of the laboratory testing equipment for *in situ* FTIR test.

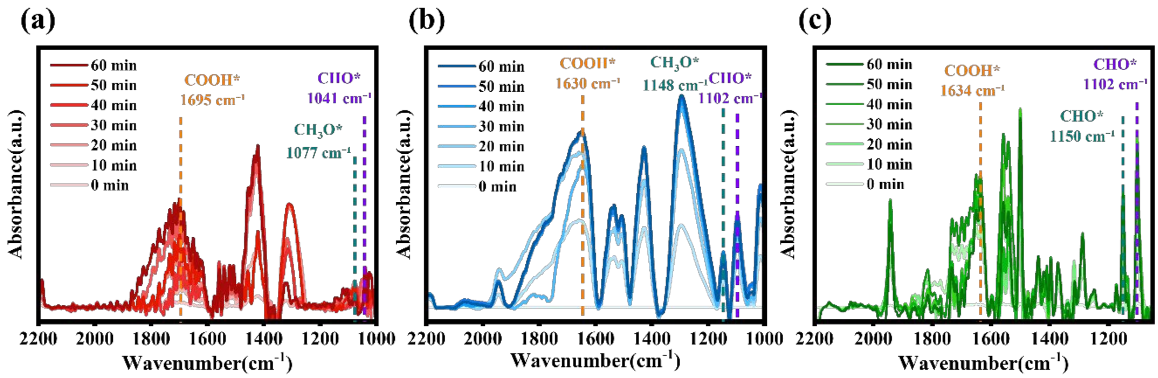

Fig. S6 The time-dependent intensity of (a) CoAl-LDH, (b)20-NiL and (c)Ni-MOF-74 for *in situ* FTIR.

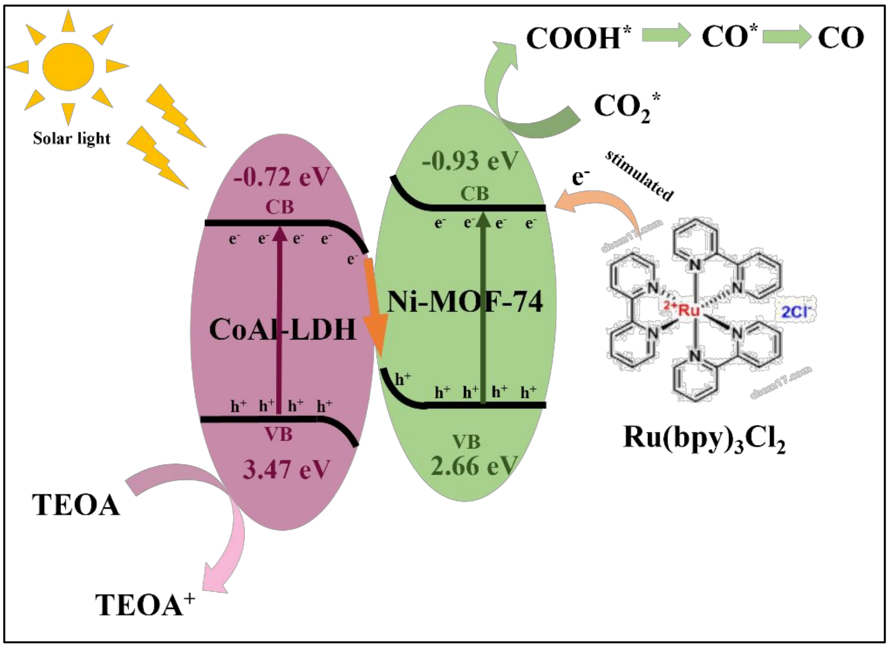

Fig. S7 Schematic illustration of the charge-transfer pathway and band-bending in a Z-scheme heterojunction photocatalyst.

Table S1 Specific surface area, pore volume and pore diameter of Ni-MOF-74, CoAl-LDH, and 20-NiL

Samples	SBET ($m^2 \cdot g^{-1}$)	Pore volume ($cm^3 \cdot g^{-1}$)	Average pore diameter (nm)
**Ni-MOF-74**	2.9484	0.0196	26.6201
**CoAl-LDH**	21.8206	0.1305	23.9303
**20-NiL**	35.4989	0.2345	26.4180

Table S2 Elemental content of 20-NiL calculated by EDS analysis.

Element	Wt%	Atomic%
C	52.87	69.72
O	23.63	23.39
Co	20.53	5.52
Al	1.79	1.05
Ni	1.17	0.32

Table S3 Comparison of photoreduction performance of different photocatalysts.

Catalyst	Photosensitizer	Sacrificial agent	Condition	Evolution ($\mu mol \cdot h^{-1} \cdot g^{-1}$)	Ref
CoAl-LDH@Ni-MOF-74	$Ru(bpy)_3{}^{2+}$	TEOA	300 W Xe	CO: 79.9	This work
$Co_1Ag_{(1+n)}$- PCN	$Ru(bpy)_3{}^{2+}$	TEOA	300 W Xe	CO: 46.82	(1)

NiAl-275	$Ru(bpy)_3^{2+}$	TEOA	300 W Xe (400 ≤ λ ≤800 nm)	CH4: 95	(2)
P25@CoAl-LDH	/	/	300 W Xe	CO: 2.21	(3)
MgAl-LDO/TiO2	/	/	450 W Xe	CO: 1.5	(4)
CN/NiFe-LDH	/	/	300 W Xe	CO: 55.1	(5)
CoAl-LDH/Ce-O_2 /RGO	/	/	(UV) light (200 W)	CO: 5.5	(6)
g-C3N4/NiAl-LD H	/	/	300 W Xe	CO:8.2	(7)
HT150MgAlTi-L DH	/	/	400 W UV(200-1000nm)	CO: 10	(8)

REFERENCES

(1) Deng A, Zhao E, Li Q, et al. Atomic cobalt–silver dual-metal sites confined on carbon nitride with synergistic Ag nanoparticles for enhanced CO_2 photoreduction[J]. ACS nano, 2023, 17(12): 11869-11881.

(2) Wang Z, Xu S M, Tan L, et al. 600 nm-driven photoreduction of CO_2 through the topological transformation of layered double hydroxides nanosheets[J]. Applied Catalysis B: Environmental, 2020, 270: 118884.

(3) Kumar S, Isaacs M A, Trofimovaite R, et al. P25@ CoAl layered double hydroxide heterojunction nanocomposites for CO_2 photocatalytic reduction[J]. Applied Catalysis B: Environmental, 2017, 209: 394-404.

(4) Zhao C, Liu L, Rao G, et al. Synthesis of novel MgAl layered double oxide grafted TiO_2 cuboids and their photocatalytic activity on CO_2 reduction with water vapor[J]. Catalysis Science & Technology, 2015, 5(6): 3288-3295.

(5) Zhu B, Xu Q, Bao X, et al. Highly selective CO_2 capture and photoreduction over porous carbon nitride foams/LDH monolith[J]. Chemical Engineering Journal, 2022, 429: 132284.

(6) Li Z, Liu Z, Li Y, et al. Flower-like CoAl layered double hydroxides modified with CeO_2 and RGO as efficient photocatalyst towards CO_2 reduction[J]. Journal of Alloys and Compounds, 2021, 881: 160650.

(7) Tonda S, Kumar S, Bhardwaj M, et al. g-C_3N_4/NiAl-LDH 2D/2D hybrid heterojunction for high-performance photocatalytic reduction of CO_2 into renewable fuels[J]. ACS Applied Materials & Interfaces, 2018, 10(3): 2667-2678.

(8) Zhao H, Xu J, Liu L, et al. CO_2 photoreduction with water vapor by Ti-embedded MgAl layered double hydroxides[J]. Journal of CO_2 Utilization, 2016, 15: 15-23.
